# Mindfulness-Based Stress Reduction Increases Mental Wellbeing and Emotion Regulation During the First Wave of the COVID-19 Pandemic: A Synchronous Online Intervention Study

**DOI:** 10.3389/fpsyg.2021.720965

**Published:** 2021-11-11

**Authors:** Maya Sanilevici, Omer Reuveni, Shahar Lev-Ari, Yulia Golland, Nava Levit-Binnun

**Affiliations:** ^1^Sagol Center for Brain and Mind, Baruch Ivcher School of Psychology, Interdisciplinary Center Herzliya (IDC), Herzliya, Israel; ^2^Department of Health Promotion, Faculty of Medicine, School of Public Health, Tel-Aviv University, Tel-Aviv, Israel

**Keywords:** COVID-19, mindfulness, MBSR, online, stress, emotion regulation, anxiety, internet-based intervention

## Abstract

The COVID-19 pandemic imposed extreme living conditions of social distancing, which triggered negative mental health problems and created challenges in seeking mental health support. Mindfulness-based interventions (MBIs) have been found to enhance wellbeing and mental health by reducing stress and anxiety and improving emotion regulation. Preliminary evidence suggests that online, synchronous MBIs may produce beneficial effects similar to face-to-face programs. However, the effectiveness of such online-MBIs to support mental health in highly stressful times, such as a global pandemic, requires further study. To this end, we investigated the effect of an online 8-week Mindfulness-Based Stress Reduction (MBSR) program on aspects of mental health during the first wave of the COVID-19 pandemic. Participants (*N*=92) who expressed interest in discounted online-MBSR programs were recruited for the study. The division into experimental and control groups was based on actual enrollment to the courses. Those who enrolled in a program were assigned to the experimental condition and those who decided not to enroll served as controls. Participants were assessed pre-intervention, post-intervention, and 1-month post-intervention for levels of mindfulness, perceived stress, anxiety, emotion regulation, and intolerance of uncertainty. Differences between the groups were tested using the general linear mixed effects model (GLMM) and Individual Growth Curve Models (IGCM) in intent to treat analysis. The findings indicated that, relative to the control group, MBSR improved mindfulness abilities (*p* <0.001), decreased anxiety (*p* <0.001), and stress (*p* <0.001) and increased emotion regulation (*p* <0.001). These effects were found to persist 1 month after the end of the program, despite the increased governmental public-health restrictions due to COVID-19 at that time. The ability to tolerate uncertainty, a central characteristic of the pandemic, was not found to be affected by the program. A mediation analysis revealed that the effect of the intervention on mental health improvement was partially mediated by the improvement in emotion regulation. Overall, the findings provide positive evidence for the feasibility of an online-MBSR program to support the mental health of individuals from the general population through the mediation of emotion regulation in challenging times, such as a global pandemic.

## Introduction

The coronavirus (COVID-19) first appeared in China at the end of 2019 and evolved into a global crisis with a significant negative impact on the physical and mental health of individuals across the globe (Benke et al., 2020; Huang and Zhao, 2020). In an effort to limit the spread of the Sars-COV-2 virus, governments worldwide imposed different forms of public-health measures, including physical distancing recommendations, closing non-essential institutions, home quarantine, curfews, and lockdowns (Lai et al., 2020). Along with the risk of a potentially life-threatening COVID-19 infection, rising unemployment, and loss of social connections, many individuals experienced significant disruptions in their everyday lives. This, in turn, impacted their ability to self-regulate and cope with the rapidly changing situation (Polizzi et al., 2020). These extreme conditions triggered feelings of uncertainty, helplessness, hopelessness, fear, and anxiety in the general population (Asmundson and Taylor, 2020; Belen, 2020; Mertens et al., 2020; Polizzi et al., 2020; Zhang et al., 2020), thus increasing the risk of developing mental health conditions (Holmes et al., 2020).

The extreme societal conditions of social distancing and stay-at-home orders not only triggered negative mental health problems (Twenge and Joiner, 2020) but also created challenges in seeking mental health support. Under these stringent restrictions, many forms of mental health support services that typically take place face-to-face could not operate. Consequently, many support services and programs were forced to rapidly shift to online platforms and develop new methods to assist their clients (Barak et al., 2009; Wind et al., 2020). The present study examined the mental health effects of a mindfulness-based program administered online during the first wave of the COVID-19 pandemic, with minor adaptations from the face-to-face version.

Mindfulness is a psychological construct drawn from the Buddhist tradition. It refers to a self-regulated, attentional stance oriented toward the present-moment experience characterized by curiosity, openness, and acceptance (Dahl et al., 2015). Mindfulness-based interventions (MBIs) cultivate mindfulness by developing skills to deal with negative thoughts and emotions in an adaptive and flexible manner (Baer, 2003; Shapiro et al., 2006; Chambers et al., 2009). These skills are believed to reduce the impact of negative emotions and free up cognitive resources to assess, observe, and give meaning to difficult circumstances, which can contribute to the development of personal goals (Coffey and Hartman, 2008; Polizzi et al., 2020). In the last few decades, mindfulness-based programs and platforms have become widespread as self-help preventive interventions for non-clinical individuals seeking to alleviate high levels of personal stress and anxiety. Additionally, such programs are considered particularly relevant in times of crisis (Behan, 2020).

Studies conducted prior to the COVID-19 pandemic have suggested that 8-week mindfulness programs, such as the standardized Mindfulness-Based Stress Reduction (MBSR; Kabat-Zinn, 2003) or the Mindfulness-Based Cognitive Therapy (MBCT; Kaviani et al., 2012) programs, can have positive consequences for psychological health and resilience. Furthermore, they have been found to produce positive benefits regarding the reduction of stress and anxiety (Semple et al., 2005; Marchand, 2012; Gu et al., 2015; Papenfuss et al., 2021). In previous studies, it has also been found that these effects can last up to several months after the end of the program (Khoury et al., 2015; Querstret et al., 2018). Various mechanisms have been proposed to account for the beneficial effects of mindfulness on mental wellbeing (Brown et al., 2007; Baer, 2009; Hölzel et al., 2011). These include an increase in emotion regulation (Garland et al., 2011; Desrosiers et al., 2013; Roemer et al., 2015; Watford and Stafford, 2015; Alkoby et al., 2018), attention regulation (Shapiro et al., 2006), decentering (Fresco et al., 2007), reperceiving (Shapiro et al., 2006), and body awareness (Hölzel et al., 2011). Recently, there have also been initial investigations of the effects of mindfulness on the ability to tolerate uncertainty (Kraemer et al., 2016; Papenfuss et al., 2021). The intolerance of uncertainty was defined as “an individual’s dispositional incapacity to endure the aversive response triggered by the perceived absence of salient, key, or sufficient information, and sustained by the associated perception of uncertainty” (Carleton, 2016).

Notably, the beneficial effects of MBIs on mental wellbeing have primarily been reported for face-to-face programs. However, some of these effects have also been replicated to a certain extent with online mindfulness programs (Aikens et al., 2014; Spijkerman et al., 2016; Krägeloh et al., 2019). A recent review and meta-analysis of the effects of MBSR/MBCT conducted *via* Group Video-conferencing (VC; Moulton-Perkins et al., 2020) identified 10 empirical studies suggesting that online-MBSR/MBCT is not inferior to traditional face-to-face MBCT/MBSR. Despite the limited evidence, these results lend weight to the feasibility and acceptability of MBCT/MBSR-VC and found similar effects to in-person interventions.

The positive effects of mindfulness practices and the growing understanding of their underlying beneficial mechanisms have pointed to mindfulness as a key protective factor for buffering the psychological impact of the COVID-19 pandemic and increasing coping abilities during these uncertain times (Behan, 2020; Hg, 2020; Vatansever et al., 2021). Dispositional mindfulness has been found to mediate the association between the fear of COVID-19 and depression and anxiety (Belen, 2020). It has also been related to enhanced wellbeing and reduced psychological distress associated with the pandemic (Conversano et al., 2020; Majeed et al., 2020; Wielgus et al., 2020). In addition, daily mindfulness practice was found to buffer the effect of COVID-19 on anxiety and sleep duration (Zheng et al., 2020). To date, only two MBIs studies have been conducted during COVID-19. The first (Lim et al., 2020) investigated an online synchronous MBSR course compared to a face-to-face MBSR course conducted during the pandemic. The results reported positive effects in reducing stress but not in the improvement of sleep quality for both groups. The second study (Zhang et al., 2021) tested the feasibility and efficacy of a 13-day online mindfulness-based intervention (composed of a 2-h training/psycho-education mindfulness session, followed by 13days of group-supported mindfulness practice). The findings indicated that there were positive effects in reducing psychological distress and anxiety symptoms.

**Figure 1:**
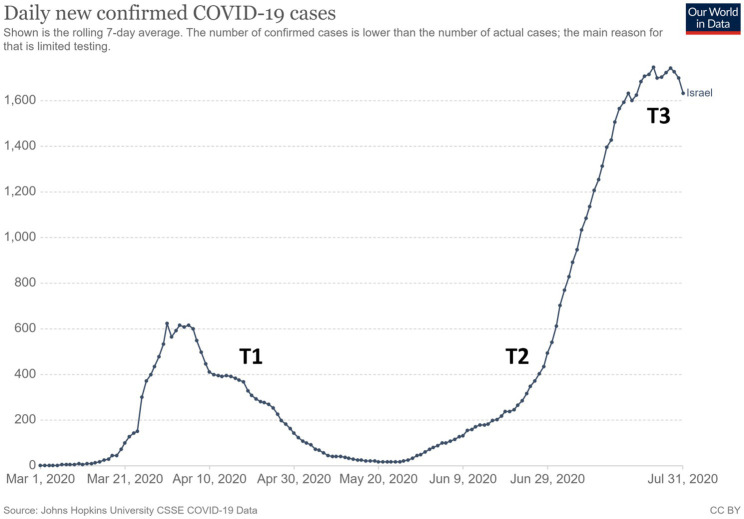
Daily changes in confirmed COVID-19 cases during the time of the study (extracted from Ritchie et al., 2020). The study’s three time points are marked along the timeline.

The present study aimed to investigate the effect of an online-MBSR program among persons seeking online services on measures of mental wellbeing, specifically stress and anxiety. The effect of online-MBSR on emotion regulation and intolerance of uncertainty was also assessed as these are highly relevant factors for managing the stress and anxiety evoked by the life-impacting challenges of COVID-19 (Freeston et al., 2020; Rettie and Daniels, 2020; Zheng et al., 2020). Therefore, an increase in emotion regulation and a decrease in intolerance of uncertainty were both suggested as mechanisms by which MBIs would exert their beneficial effects. The online-MBSR programs were provided by a mindfulness center associated with the university (The Interdisciplinary Center Herzliya). The programs were initiated in mid-April 2020, less than 2 months after the first case of COVID-19 was detected in Israel. This occurred 4–6weeks after the government introduced the first series of COVID-19 public-health measures, including physical distancing regulations, home quarantine, the closing of non-essential institutions, curfews, and lockdowns (see Figure 1 for details). When the courses began (T1), these measures were only beginning to be withdrawn. Both programs ended after 2 months (T2), a time characterized by a slow rise in COVID-19-related cases and deaths and governmental threats that public-health measures will be activated again. A month later (T3), COVID-19-related cases and deaths had risen sharply, non-essential institutions were closed again, and social distancing was enforced, thus limiting social interactions to groups of up to 10 people.

Based on previous research regarding face-to-face and online-MBSR courses, it was hypothesized that online-MBSR would reduce perceived stress and anxiety, as well as intolerance to uncertainty, and increase emotion regulation abilities. It was further hypothesized that these measures would stay relatively unchanged for the control group. In addition, it was expected that the MBSR benefits would also remain evident for 1 month after the end of the course.

## Materials and Methods

### Participants

The study was a non-randomized controlled trial, using a convenience sample. Participants (*N*=92) who enrolled in two online synchronous MBSR courses provided by a mindfulness center affiliated with the university (Muda Institute for Mindfulness, Science and Society) were recruited to participate as the intervention group in the study (MBSR group). Due to the increase in economic uncertainty following the lockdown and governmental restrictions, participants could pay the registration fee according to a sliding scale. The sliding scale prices were between 15 and 60% less than the pre-COVID-19 face-to-face MBSR courses. Before final payment, participants were informed that the online-MBSR courses would be assessed by researchers and that they would be approached and asked to participate in the study. However, it was made clear that participation in the study was voluntary and would not affect their experience in the course in any way.

Participants in the control group were recruited by approaching individuals who expressed interest in these online courses but eventually did not enroll due to personal reasons or because the program was already full. In exchange for participating in the study, they were eligible to enter a raffle for 10 mindfulness books and two vouchers for participation in later MBSR courses at the end of the study. Participants who agreed (*N*=46) were allocated to the control group. Demographic characteristics for all participants are shown in Table 1.

**Table 1:** Demographic characteristics of participants pre-intervention.

	Intervention group		Control group		Full sample		t-tests or χ^2^ two tailed
Demographic characteristics	(*N* =46)		(*N* =46)		(*N* =92)		Significance
	*N*	%	*N*	%	*N*	%	
Age in years [M (SD)]	44.37 (14.07)		39.22 (15.403)		41.79 (14.897)		*t*(90)=1.68 *p* = 0.10
Gender							*χ*^2^(2)=1.18 *p* = 0.55
Male	13	28	11	24	24	26	
Female	33	72	34	74	67	73	
Other	0	0	1	2	1	1	
Marital status							*χ*^2^(2)=7.40 *p* =0.06
Single	8	17	19	41	27	29	
In a relationship	6	13	7	15	13	14	
Married	28	61	18	39	46	50	
Divorced	4	9	2	4	6	7	
Education status (in years)							*χ*^2^(2)=1.85 *p* =0.60
Up to 12years	3	7	1	2	4	4	
12–15years	8	17	11	24	19	21	
16–18years	16	35	18	39	34	37	
Over 18years	19	41	16	35	35	38	
Religious status							*χ*^2^(2)=1.07 *p* =0.79
Secular	39	89	39	85	78	87	
Traditional	4	9	5	11	9	10	
Religious	1	2	1	2	2	2	
Orthodox	0	0	1	2	1	1	
Living situation							*χ*^2^(2)=4.57 *p* =0.47
Living alone	6	13	8	17	14	15	
Roommates	2	4	6	13	8	9	
Parents	5	11	5	11	10	11	
Significant other	18	39	16	35	34	37	
Significant other and children	13	28	11	24	24	26	
Only with children	2	4	0	0	2	2	
Employment status							*χ*^2^(2)=4.20 *p* =0.24
Working	26	57	31	67	57	62	
Unpaid vacation	5	11	8	17	13	14	
Unemployed	10	22	4	9	14	15	
Other^a^	5	11	3	7	8	9	

*N* =92 apart from religious status for which *N* =90 (control *n* =46, MBSR *n* =44).

a Other types of working status, such as pension and sick leave.

### Procedure

This study was approved by the IDC Research Ethics Board (1920501_P). All participants who agreed to participate in the study signed a consent form. They then received a link to an online survey with demographic questions and self-report measures to assess mindfulness, state anxiety, perceived stress, difficulties in emotion regulation, intolerance of uncertainty, and loneliness (pre-intervention: T1). Immediately after the MBSR group completed their MBSR programs (post-intervention: T2) and 1 month later (1-month post-intervention: T3), all participants were administered the same survey without the demographic questions. The overlap of the study’s data acquisition time points with the COVID-19 illness rate is depicted in Figure 1 (based on Ritchie et al., 2020). Overall, 71 participants completed all three measurements between the control (*N*=38) and intervention (*N*=33) groups.

#### MBSR Intervention

The course followed the standardized MBSR protocol developed by Kabat-Zinn (2013), which is designed to guide participants in practicing, integrating, and applying mindfulness in their everyday lives. The MBSR protocol was administered in an 8-week group structured intervention with weekly 2.5-h meetings. The entire program was administered online using the Zoom platform. Apart from the weekly meetings, the intervention protocol also included assignments for daily home practice (30min per day) accompanied by guided meditation recordings and a 4-h long silent retreat during the 6th week of the program. In each class, participants learned about and practiced different forms of mindfulness. This included formal practices of yoga, sitting meditation, body scan, and walking meditation, as well as informal practices, such as mindful eating, speaking and listening, and mindfulness of daily activities. For example, in the body scan practice (Kabat-Zinn, 1990, p. 75–93; Segal et al., 2013, p. 110–117), participants learned to mobilize their attention sequentially from body part to body part while increasing sensitivity to the inner bodily sensations. In sitting practices, participants cultivated the ability to bring stable attention and awareness to bodily (e.g., the breath) and mental phenomena that constantly enter one’s stream of consciousness (Kabat-Zinn, 1990, p. 59–74; Segal et al., 2013, p. 146–147, 164–165). This was done to cultivate receptivity and willingness to stay in contact with all aspects of the experience (Kabat-Zinn, 1990, p. 59–74; Segal et al., 2013, p. 146–147, 164–165). A central component in these practices is the cultivation of awareness to subtle thoughts, sensations, feelings, shifts in affective tone, and incoming sensory information (Jha et al., 2007; Dahl et al., 2015), which lead to increased body awareness and sensitivity to emergent affective cues in the experiential field (Carmody et al., 2009; Cebolla et al., 2016). These various practices have been found to increase executive control and cognitive flexibility (e.g., Moore and Malinowski, 2009) and decrease automatic responses to emotional experiences (e.g., Jha et al., 2007). The current study assessed two online-MBSR interventions, each led by a certified MBSR instructor with over 20years of personal mindfulness practice and more than 3years of experience teaching the MBSR protocol. Adjustment to the online format included as: (1) The silent retreat, which is usually 8h long, was shortened to 4h and included less mindful movement practices; (2) Two short breaks were usually provided during the 2.5-h weekly meetings instead of one long break; and (3) One teacher used PowerPoint slides for the sections on psycho-education.

### Measures

#### Mindfulness

Mindfulness was assessed using the *Freiburg Mindfulness Inventory* (FMI; Walach et al., 2006), which is a self-report measure consisting of 14 items that measure trait-mindfulness (e.g., “I am open to the experience of the present moment”). Participants are asked to refer to the last 14days when responding to the items. Items are rated on a 4-point Likert scale ranging from 1 (“Rarely”) to 4 (“Almost always”), with higher scores indicating higher mindfulness levels. In the present study, this measure demonstrated high internal consistency across all three measurements (Mean Cronbach’s α=0.856).

#### State Anxiety

State anxiety was measured using the *State-Trait Anxiety Inventory* (STAI; Spielberger, 2010). This self-report measure consists of two subscales examining both state and trait anxiety. For this study, only the state subscale was examined due to considerations regarding the study’s length and the other measures involved. The state anxiety subscale consists of 20 items referring to participants feelings *right now* (e.g., “I feel frightened”) rated on a 4-point Likert scale ranging from 1 (“Not at all”) to 4 (“Very much so”) with higher scores indicating higher levels of anxiety. In the present study, this measure demonstrated high internal consistency across all three measurements (Mean Cronbach’s α=0.939).

#### Perceived Stress

Perceived stress was measured using the *Perceived Stress Scale* (PSS; Cohen et al., 1983), which is a 14-item self-report scale rated on a 5-point Likert scale ranging from 0 (“Never”) to 4 (“Very often”). Participants are asked to indicate how often they felt a certain way (e.g., “In the last month, how often have you been upset because of something that happened unexpectedly?”). In the present study, this measure demonstrated high internal consistency across all three measurements (Mean Cronbach’s α=0.864).

#### Emotional Regulation

Emotional regulation was assessed using the *Difficulties in Emotional Regulation Scale* (DERS), which is an 18-item self-report measure (Victor and Klonsky, 2016) based on the original version (Gratz and Roemer, 2004). Each item is rated on a 5-point Likert scale ranging from 1 (“Almost never (0–10%)”) to 5 (“Almost always (91–100%)”). This measure consists of one total score that measures emotion regulation difficulties, with higher scores showing higher emotional dysregulation (e.g., “When I am upset, I become embarrassed for feeling that way”). In the present study, the measure demonstrated high internal consistency across all three measurements (Mean Cronbach’s α=0.885).

#### Ability to Tolerate Uncertainty

Ability to tolerate uncertainty was measured with the *Intolerance of Uncertainty Scale-Short Version* (IUS12; Carleton et al., 2007), based on the original 27-item version (Freeston et al., 1994). Items are rated on a 5-point Likert scale ranging from 1 (“Not at all characteristic of me”) to 5 (“Entirely characteristic of me”), with higher scores indicating greater difficulties dealing with uncertainty. The scale is constructed on two dimensions: prospective anxiety (e.g., “Unforeseen events upset me greatly”) and inhibitory anxiety (e.g., “Uncertainty keeps me from living a full life”). In the present study, the measure’s total score demonstrated high internal consistency across all three measurements (Mean Cronbach’s α=0.883) as well as high internal consistency for both factors: prospective anxiety (Mean Cronbach’s α=0.802) and inhibitory anxiety (Mean Cronbach’s α=0.818).

### Statistical Analysis

An intention-to-treat analysis approach was implemented for this trial and all participants were included in the data. Group differences at T1 were tested using t-test and χ^2^ analyses. Pearson correlations were used for the correlation matrix of the different measures.

Differences between the groups were tested using the general linear mixed effects model (GLMM). The dependent variables were the manipulation check (the mindfulness measure) and the intervention outcome measures (perceived stress, state anxiety, emotion regulation difficulties, and intolerance of uncertainty).

Time of the measurement, intervention group, and the interaction of time X group were entered as fixed factors with random intercepts of subjects. The main effect of interest in the study was the interaction effect of time and group. Prior to conducting these analyses, Little’s test was applied to ensure the assumption for missing completely at random (MCAR) was met. Missing data were handled with a full information maximum likelihood (FIML) method (Heck et al., 2013). Bonferroni adjustments were made to correct for multiple comparisons. These analyses were conducted using SPSS 25 (IBM, New York City).

**Figure 2:**
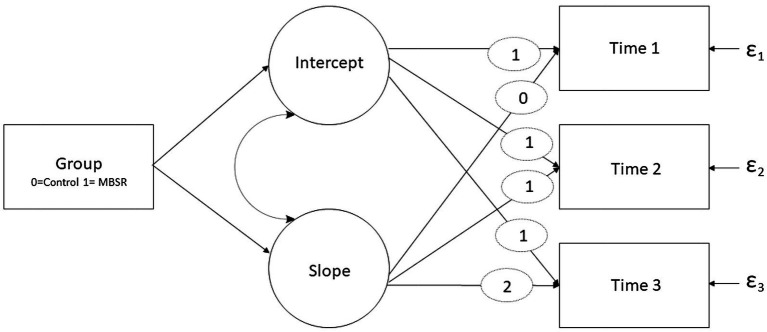
An illustration of the individual growth curve model. The rectangles represent observed variables, while the circles represent latent factors. Curved arches represent correlations and ε represents residual random parts. Fixed weights for intercept and time weight for slopes are represented by ellipses.

Following this analysis, an individual growth curve modeling (IGCM) framework was added. This was done to estimate individual changes over time (Hoffman, 2020) in which latent growth parameters of intercept (i.e., mean starting point) and slope (i.e., rate of change) were examined. Using Mplus V. 8.3.1, the slope was modeled on linear time points entered as 0 (T1), 1 (T2), and 2 (T3). Additionally, group was added as a time invariant covariate and coded as 0 (control) and 1 (intervention) to assess the covariance of group with the intercept and time slopes. The residual random parts were also entered (see Figure 2 for the model diagram). Bayesian (BIC) criteria were used for model fit with 5,000 iterations and 95% bias-corrected confidence intervals.

Mediation analyses were conducted using Hayes, 2017 PROCESS macro for SPSS 25 (version 3.5, model 4; with 5,000 bootstrap iterations and 95% bias-corrected confidence intervals). A variance inflation factor (VIF) was calculated for the independent variables to take into account the presence of multicollinearity, of which a VIF larger than 5 is suggested to detect multicollinearity (Copeland, 1997).

## Results

Sample characteristics A total of 92 participants participated in the study; most of the participants (73%) were female. Sixty-four percent of the participants were either married or in a relationship, with only 15% reporting living alone. Twenty-nine percent of the participants reported being financially affected by COVID-19-related consequences and were either fired or on unpaid leave. The vast majority had at least one academic degree (96%) and was secular (87%). All sample characteristics are shown in Table 1.

### Group Differences at T1

Despite the lack of randomization, the intervention and control groups did not exhibit significant differences in demographic characteristics (Table 1). At T1 (before the intervention), participants who enrolled in the MBSR courses, compared to controls, exhibited lower scores on the mindfulness scale [*t*(91)=−3.709, *p* <0.001] and higher scores on difficulties in emotion regulation scale [*t*(90)=2.054, *p* = 0.043]. The rest of the dependent variables did not differ significantly at T1 (perceived stress scale [*t*(90)=1.91, *p* = 0.059]; state anxiety scale [*t*(90)=1.53, *p* = 0.129]; and intolerance of uncertainty [*t*(90)=1.22, *p* = 0.224]).

### Analysis of Intercorrelations

Correlations between mindfulness, mental wellbeing, emotion regulation, and intolerance of uncertainty were calculated using Pearson correlations with all tested variables at T1 (see Table 2). As hypothesized, significant correlations between all tested variables were found, which is in line with previous reports (Baer, 2009; Alkoby et al., 2018; Papenfuss et al., 2021).

**Table 2:** Correlations for study-dependent variables at T1.

S. No.	Variable	1	2	3	4	5
1.	Mindfulness	—				
2.	Perceived stress	−0.600^***^	—			
3.	State anxiety	−0.509^***^	0.651^***^	—		
4.	Difficulties in emotion regulation	−0.646^***^	0.695^***^	0.547^***^	—	
5.	Intolerance of uncertainty	−0.495^***^	0.485^***^	0.344^**^	0.534^***^	—

** *p* =0.003;

*** *p* <0.001.

### Manipulation Check: Effect of Online-MBSR on Mindfulness

A significant fixed effect of time was detected when applying the GLMM model to reported mindfulness scores [*F*(2, 244)=21.129, *p* <0.001]. In addition, pairwise comparisons *via* Bonferroni adjustments revealed significant increases between T1 and T2 (*p* <0.001), as well as T1 and T3 (*p* <0.001), but not for T2 and T3. A significant fixed effect of group was also found [*F*(1, 244)=5.799, *p* =0.017], with the control group being higher than the MBSR group. In line with the expectation that the online-MBSR program would affect mindfulness levels (manipulation check), a significant group X time interaction was found [*F*(2, 244)=4.868, *p* =0.008; see Table 3]. Pairwise comparisons with Bonferroni adjustment revealed that, while there were no significant changes over time in the control group, the MBSR group revealed significant differences in mindfulness scores, which increased from T1 to T2 [*t*(244)=−6.105, *p* <0.001]. The difference also remained significantly greater at the 1-month follow-up (difference between T1 and T3, [*t*(244)=−5.604, *p* <0.001]. No significant changes were found between T2 and T3 within the MBSR group.

**Table 3:** Means, standard deviations, and one-way analyses of variance interaction effects of online-MBSR.

Measure	Pre-intervention		Post-intervention		1-month post		Group X Time	
	*M*	*SD*	*M*	*SD*	*M*	*SD*	*F*(2,244)	*p*
Mindfulness							4.868	0.008
MBSR	30.80	5.39	35.68	5.65	35.50	4.74		
Contr	35.30	6.54	36.98	6.91	37.51	6.72		
Perceived stress							13.341	<0.001
MBSR	35.39	5.85	28.49	6.06	28.91	5.17		
Control	32.72	7.48	30.76	6.88	30.88	6.69		
State Anxiety							4.496	0.012
MBSR	45.63	11.12	37.16	10.57	38.41	9.03		
Control	42.09	11.03	39.38	12.16	41.46	13.08		
Emotional regulation difficulties							9.984	<0.001
MBSR	43.26	11.63	35.51	8.84	35.76	8.85		
Control	38.39	11.10	37.27	10.90	37.20	10.65		
Intolerance of uncertainty							0.731	0.731
MBSR	32.45	8.57	31.62	8.08	30.32	7.07		
Control	30.24	8.80	29.87	8.91	28.88	8.75		

The IGCM analysis (see Table 4) found a significant group effect on the slope changes, indicating that the MBSR group demonstrated higher rates of change in mindfulness over time (*β*=0.41, 95% CI: 0.08–0.87). A significant group effect on the starting point was also found, with the MBSR group having a lower score at the starting point (*β*=−0.38, 95% CI: −0.55−−0.16). However, significant coefficients for the slope and intercept were not found, indicating that the lower mindfulness levels at the starting point did not affect the rate of change.

**Table 4:** Standardized estimates, standard errors, and significance in individual growth curve models.

Measure	Mindfulness	Perceived stress	Difficulties in emotion regulation	State anxiety
	Estimate (SEM)	Estimate (SEM)	Estimate (SEM)	Estimate (SEM)
Intercept	6.08 (0.66)^*^	5.48 (0.68)^*^	3.79 (0.46)^*^	4.38 (0.70)^*^
Slope	0.47 (0.41)	−0.35 (0.29)	−0.19 (0.34)	0.01 (0.27)
Intercept variance	0.86 (0.07)^*^	0.97 (0.04)^*^	0.97 (0.04)^*^	0.97 (0.05)^*^
Slope variance	0.83 (0.19)^*^	0.83 (0.21)^*^	0.67 (0.22)^*^	0.80 (0.19)^*^
Intercept X Group^a^	−0.38 (0.10)^*^	0.17 (0.12)	0.18 (0.11)	0.18 (0.12)
Slope X Group	0.41 (0.20)^*^	−0.67 (0.16)^*^	−0.58 (0.20)^*^	−0.45 (0.19)^*^
Intercept X Slope	−0.21 (0.32)	−0.13 (0.36)	−0.35 (0.32)	−0.27 (0.36)

* *p* <0.05 two tailed.

a Group variable is coded as 0=control, 1=MBSR group.

### Effect of Online-MBSR on Mental Health Indicators

The effect of online-MBSR on mental health was examined by analyzing the effects on perceived stress and state anxiety using GLMM analysis (Table 3). A fixed effect of time was found for both perceived stress [*F*(2, 243)=36.892, *p* <0.001] and state anxiety [*F*(2, 243)=10.595, *p* <0.001]. In both measures, the same pattern for mindfulness scores emerged with significant differences between T1 and T2 (*p* <0.001), as well as T1 and T3 (*p* <0.001 for perceived stress and *p* =0.023 for state anxiety). For both measures, no significant effects were found for group (perceived stress: *p*=0.603; state anxiety: *p*=0.876). Central to the study’s hypothesis, a significant group X time interaction was found for both perceived stress [*F*(2, 244)=13.341, *p* <0.001] and state anxiety [*F*(2, 244)=4.496, *p* =0.012; see Table 3]. Pairwise comparisons with Bonferroni adjustment revealed that, while the control group showed no significant changes over time in both measures, significant effects of time were found for the MBSR group. Specifically, significant reductions in perceived stress [*t*(243)=9.775, *p* <0.001] and state anxiety [*t*(243)=5.380, *p* <0.001] were found from T1 to T2. Remarkably, these reductions remained significant between T1 and T3 for both perceived stress [*t*(243)=9.775, *p* <0.001] and state anxiety [*t*(243)=3.764, *p* <0.001].

Applying the IGCM analysis (Table 4), and in line with the GLMM results, a significant group X slope coefficient was found, indicating that the MBSR group exhibited a greater change rate for the decrease in perceived stress (*β*=−0.67, 95% CI: −0.95, −0.34) and state anxiety (*β*=−0.45, 95% CI: −0.87, −0.11). No significant group X intercept coefficients were found for either measure, indicating no significant differences between groups at T1. None of the slope and intercepts interaction coefficients were found to be significant, indicating slope change rates were not affected by starting levels.

### Effect of Online-MBSR on Emotion Regulation and Intolerance to Uncertainty

The effect of online-MBSR on mechanisms associated with the beneficial outcomes of MBIs was investigated by analyzing the difficulties in emotion regulation (DER) and intolerance of uncertainty (IU) scores. For the GLMM analysis, a fixed effect of time emerged for emotion regulation [*F*(2, 243)=16.262, *p* <0.001], with the same pattern found between T1 and T2 as well as T1 and T3 (*p* <0.001), as in the previous measures. The group effect revealed no significant outcome (*p*<0.827). As hypothesized, a group X time interaction effect showed significant results for emotion regulation [*F*(2, 243)=9.984, *p* <0.001; see Table 3]. Pairwise comparisons with Bonferroni adjustment revealed the hypothesized pattern, with no significant differences in time for the control group and significant differences in time among the intervention group. Namely, the MBSR group reported improved emotion regulation, showing significant reductions in emotion regulation difficulties from T1 to T2 [*t*(243)=5.952, *p* <0.001]. These differences remained significant at T3 [*t*(243)=5.365, *p* <0.001].

These effects were corroborated in the IGCM analysis (Table 4). A significant group X slope coefficient was found for emotion regulation, indicating that the MBSR group exhibited a greater change rate for the decrease in difficulties in emotion regulation (*β*=−0.58, 95% CI: −0.93, −0.20). Contrary to the above analysis, which suggested that the MBSR group had greater difficulties in emotion regulation at baseline, no significant group X intercept coefficients were found for emotion regulation in this more advanced analysis, indicating no significant differences between groups at T1. In addition, none of the slope and intercepts interaction coefficients were found to be significant, indicating that the slope change rates were not affected by starting levels.

**Figure 3:**
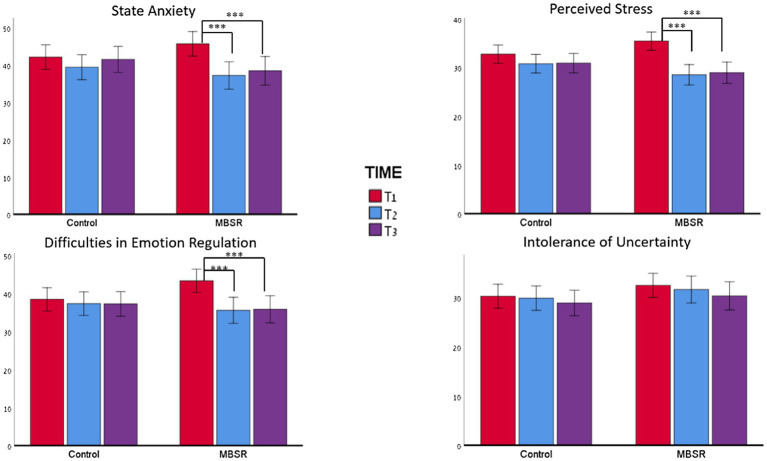
Changes in outcomes across the three time points for state anxiety, emotion regulation, and intolerance of uncertainty. For all measures, no effect of time was observed for the control group. However, in the MBSR group, for all measures except the IUS12, a significant difference was found between T1 and T2 and between T1 and T3. ^***^p<0.001.

For intolerance of uncertainty, no significant fixed effects were found in the GLMM analysis for time (*p*<0.317), group (*p*<0.266), or the group X time interaction (*p*<0.731, see Table 3). Furthermore, no effects were found in the IGCM analysis (Figure 3).

### Emotion Regulation as a Mediator Between MBSR and Improvement in Mental Health

**Figure 4:**
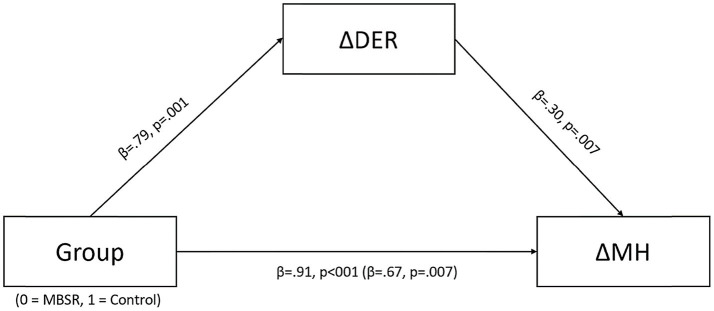
Mediation of intervention group on changes in mental health through changes in emotion regulation (the models present standardized beta coefficients). ^a^∆DER=difficulties in emotion regulation measured at T3 – difficulties in emotion regulation measured at T1/difficulties in emotion regulation measured at T1. ^b^∆MH=the combined standardized score of state anxiety and perceived stress at T3 – the combined standardized score of state anxiety and perceived stress at T1.

Given these positive findings, emotion regulation (DER) at T3 compared to T1 was examined to determine whether fewer emotion regulation difficulties mediated the effects of MBSR on mental health indicators (MH). For this aim, a mediation analysis was conducted. The model was specified with the independent variable defined as group (MBSR=0, control=1). The mediating variable was defined as the difference in DERS between T3 and T1. Due to initial group differences found in the t-test conducted at T1, the differences between T3 and T1 were normalized by the value at T1 (∆DER=(DER_T3_−DER_T1_)/DER_T1_), such that negative scores indicated fewer emotion regulation difficulties post-intervention. The dependent variable was mental health indicators. The anxiety and stress scale sums were standardized and a mean score was computed for both measures to achieve the MH score. Multicollinearity between predictors was not found [VIF=1.19]. In the model, the dependent variable was defined as the difference in MH between T3 and T1 (∆MH=MH_T3_−MH_T1_), with negative scores indicating less stress and anxiety. The total effect of the model (*β*=0.91, 95% CI: 0.387, 1.042, *t*=4.346, *p*<0.001) was reduced when the mediator variable was added (*β*=0.67, 95% CI: 0.362, 2.258, *t*=2.757, *p*=0.007). Zero was not included in the indirect effect (*β*=0.24, 95% CI: 0.717, 0.453), indicating a significant mediation of MBSR on mental health through emotion regulation (Figure 4).

## Discussion

The current study investigated the effects of an online synchronous MBSR course during the first 4 months of the global COVID-19 pandemic. This period was characterized by high levels of uncertainty, unemployment, perceptions of direct threat, and loss of social support due to social distancing regulations (Asmundson and Taylor, 2020; Belen, 2020; Mertens et al., 2020; Polizzi et al., 2020). These extreme conditions not only increased the risk for the development of mental health conditions (Holmes et al., 2020; Twenge and Joiner, 2020) but also created challenges in seeking mental health supports (Wind et al., 2020). Specifically, an online-MBSR was examined to determine whether it would reduce stress and anxiety, improve the ability to employ emotion regulation in difficult emotional states and reduce intolerance of uncertainty – two mechanisms considered to underlie the beneficial effects of MBIs.

The findings indicated that, relative to the control group, the online-MBSR improved the mindfulness abilities of the participants, which served as the manipulation check. The online-MBSR led to decreased anxiety, stress, and emotion regulation difficulties. These effects persisted 1 month after the end of the program (T3), despite the pandemic being worse at that time relative to T1 and T2. As suggested by the IGCM analyses, these changes were related to the effect of the MBSR intervention and not a regression to the mean or initial group differences. Moreover, the decrease in mental health measures observed at T3 was mediated by improved emotion regulation. Overall, the results provide positive evidence for the feasibility of an online-MBSR program to support the mental wellbeing of individuals from the general population who seek online treatment, even in challenging times, such as the COVID-19 pandemic.

The positive effects of this study’s online-MBSR program on mental wellbeing are consistent with numerous studies conducted before the pandemic with face-to-face programs (Baer, 2003; Brown et al., 2007; Chiesa and Serretti, 2009; Querstret et al., 2018). They are also consistent with the studies that investigated online-MBSR/MBCT programs before the pandemic (Moulton-Perkins et al., 2020), as well as the few studies that took place during the pandemic (Behan, 2020; Hg, 2020; Polizzi et al., 2020; Vatansever et al., 2021).

Notably, most studies conducted during the pandemic were cross-sectional and measured whether individuals with higher trait-mindfulness were more resilient to pandemic-related stress and anxiety. As far as is known, there have only been two intervention studies that studied the effect of MBI programs during the COVID-19 pandemic (Lim et al., 2020; Zhang et al., 2021). The first (Lim et al., 2020) compared an online-MBSR program to a face-to-face MBSR program – both of which were conducted during the pandemic – as well as to a MBSR group conducted prior to the onset of the pandemic. The second study (Zhang et al., 2021) tested the feasibility and efficacy of a brief online mindfulness-based intervention that lasted 13days. In both cases, online-MBIs had positive effects in reducing psychological distress and anxiety symptoms.

The current study extends the field of online-MBSR studies in several ways. First, in the context of a highly stressful situation, such as a global pandemic, it suggests that the positive mental health effects of an online-MBSR course can be maintained, according to the findings that the effects here were evident 1 month after the end of the course. Moreover, as has been suggested for face-to-face MBSR (Hayes and Feldman, 2004; Chambers et al., 2009; Goldin and Gross, 2010; Watford and Stafford, 2015), this study highlights the role of emotion regulation in mediating the positive effects of an online-MBSR on mental health measures. As stressful events are inherently highly emotional (Southwick et al., 2011) and can increase vulnerability to psychopathology (Grueschow et al., 2021), the ability to regulate emotions may be a critically important factor in determining stress resilience (Barzilay et al., 2020; Kaldewaij et al., 2021) and mental wellbeing (Satchit et al., 2011; Silton et al., 2020). As the present study was conducted under the highly stressful conditions of a global pandemic, it could be assumed that the beneficial effects of online-MBSR on emotion regulation can increase stress resilience and reduce vulnerability to psychopathology during everyday life challenges (such as acute stress in the workplace; Grueschow et al., 2021).

High levels of uncertainty characterized the first waves of the COVID-19 pandemic. Therefore, this study also investigated the relationship between mindfulness and difficulties in tolerating uncertainty. Replicating previous findings (Kraemer et al., 2016), it was found that higher trait-mindfulness was associated with reduced intolerance of uncertainty for all participants. However, contrary to some of the other measures, there were no significant differences in intolerance of uncertainty between groups at T1 and no effect was found in the online-MBSR intervention. It is possible that the characteristics of uncertainty in the extreme period of a global pandemic are not well captured by the IUS12 scale or that they masked the effects of a short-term mindfulness practice. Future studies should develop tools to assess uncertainty in conditions that are more characteristic and sensitive to the conditions in a global pandemic.

## Limitations

There are several limitations to the current study that warrant further consideration. First, the population was not sampled randomly but rather was based on the participants’ preliminary interest in enrolling in a MBSR course during the first lockdown. This common interest in mindfulness may suggest that the sample had specific personality attributes that might have affected the outcomes and, therefore, may not necessarily reflect the general population. Second, to conduct the study in the unique conditions of the first stages of the pandemic, the allocation of control and experimental groups was not randomized due to time constraints. However, it should be noted that a comparison between the intervention and control groups did not reveal any differences between the participants’ demographic characteristics at baseline. Furthermore, a sensitivity analysis of IGCM with outcomes measures at baseline (T1) as covariates was conducted to control for possible initial differences in variables between the groups. Still, future studies should randomize participants to refute the possibility that the current findings are limited to the subset of participants who, at unpredictable times, such as a global pandemic, presented with reduced mindfulness and emotion regulation abilities and were more prone to seek and commit to self-help interventions for support. Another limitation of the study is the assessment of outcomes only a month after the end of the intervention and not at additional time points. Although positive outcomes remained high, despite the worsening of the pandemic, it is not clear whether the gains would be sustained for extended periods without further structured support (e.g., group setting and instructor). An additional possible limitation can be taken from Canby et al. (2021), who showed that instructor – and group – related factors play a role in the therapeutic effects of MBIs. Naturally, a group formed at the beginning of a global pandemic may share the common cohesive experience of difficulty that could positively contribute to the program’s overall effect. Since the MBSR group was not compared to an active control, the possibility cannot be eliminated that the positive effects found were due to the specific teachers who led the courses and/or the group dynamics. Finally, this study was based on self-report measures, which are known for their limitations (Razavi, 2001). Future studies should employ behavioral and physiological tools to study the neural mechanisms underlying the effects of online-MBIs. Based on the present study’s and others’ findings, it is expected that neural mechanisms would be associated with emotion control and regulation (Grueschow et al., 2020, 2021; Kaldewaij et al., 2021) as well as overlapping the neural mechanisms underlying face-to-face MBIs (e.g., Farb et al., 2007, 2013).

## Conclusion

Overall, the findings suggest that an online synchronous MBI can successfully reduce stress and anxiety while improving emotion regulation in participants during highly stressful conditions caused by a global pandemic. The improved outcomes continued to last 1 month after the intervention, even when the COVID-19 situation took a turn for the worse. These findings are especially meaningful given the scarce evidence (Moulton-Perkins et al., 2020) on online stress reduction interventions during COVID-19, characterized by the lack of access to face-to-face mental health support.

## Data Availability Statement

The original contributions presented in the study are included in the article/supplementary material, further inquiries can be directed to the corresponding author.

## Ethics Statement

The studies involving human participants were reviewed and approved by the Ethics Committee of Baruch Ivcher School of Psychology, The Interdisciplinary Center Herzliya. The patients/participants provided their written informed consent to participate in this study.

## Author Contributions

MS, NL-B, and YG participated in the study design. MS conducted the study. MS, NL-B, YG, SL-A, and OR were involved in the data analysis and preparation of the manuscript. All authors contributed to the article and approved the submitted version.

## Conflict of Interest

The authors declare that the research was conducted in the absence of any commercial or financial relationships that could be construed as a potential conflict of interest.

## Publisher’s Note

All claims expressed in this article are solely those of the authors and do not necessarily represent those of their affiliated organizations, or those of the publisher, the editors and the reviewers. Any product that may be evaluated in this article, or claim that may be made by its manufacturer, is not guaranteed or endorsed by the publisher.
